# Local atomic arrangements and lattice distortions in layered Ge-Sb-Te crystal structures

**DOI:** 10.1038/srep26724

**Published:** 2016-05-25

**Authors:** Andriy Lotnyk, Ulrich Ross, Sabine Bernütz, Erik Thelander, Bernd Rauschenbach

**Affiliations:** 1 Leibniz Institute of Surface Modification (IOM), Permoserstr. 15, D-04318 Leipzig, Germany

## Abstract

Insights into the local atomic arrangements of layered Ge-Sb-Te compounds are of particular importance from a fundamental point of view and for data storage applications. In this view, a detailed knowledge of the atomic structure in such alloys is central to understanding the functional properties both in the more commonly utilized amorphous–crystalline transition and in recently proposed interfacial phase change memory based on the transition between two crystalline structures. Aberration-corrected scanning transmission electron microscopy allows direct imaging of local arrangement in the crystalline lattice with atomic resolution. However, due to the non-trivial influence of thermal diffuse scattering on the high-angle scattering signal, a detailed examination of the image contrast requires comparison with theoretical image simulations. This work reveals the local atomic structure of trigonal Ge-Sb-Te thin films by using a combination of direct imaging of the atomic columns and theoretical image simulation approaches. The results show that the thin films are prone to the formation of stacking disorder with individual building blocks of the Ge_2_Sb_2_Te_5_, Ge_1_Sb_2_Te_4_ and Ge_3_Sb_2_Te_6_ crystal structures intercalated within randomly oriented grains. The comparison with image simulations based on various theoretical models reveals intermixed cation layers with pronounced local lattice distortions, exceeding those reported in literature.

Ge-Sb-Te (GST) compounds are of high interest due to their technologically outstanding optical and electronic properties. Thin films of GST alloys are widely used as phase change materials (PCMs) in optical storage media^1^,^2^,^3^,^4^ and are also major contenders for the next generation non-volatile RAM^5^,^6^,^7^,^8^. The operating principle of conventional PCM devices is based on the reversible transformation between the amorphous and metastable crystalline phases triggered either by optical or electrical ultrafast pulses. Interfacial PCMs (iPCMs)^9^ or chalcogenide superlattices consisting of Sb_2_Te_3_ and GeTe multilayers are a promising candidate for data storage devices with reduced energy consumption since reversible transition between SET and RESET states is assumed to be constrained by motion of atoms in 1D instead of 3D as in the case of conventional PCM devices. Thus, the switching mechanism of iPCMs is determined by the local atomic arrangement in distinct layers^10^,^11^,^12^,^13^, which also defines the electronic properties of the materials^14^. In particular, theoretical simulations showed that iPCMs can be a 3-D topological insulator^15^ or a Dirac semimetal^11^. Investigations of atomic structure in such technologically relevant iPCM revealed that various layered GST crystal structures can be formed during iPCMs production^16^,^17^,^18^. Consequently, the knowledge of the proper local atomic arrangement in layered GST alloys is of paramount importance in order to understand the switching mechanism of iPCMs and their material properties.

The overall structure of layered GST compounds consists of rocksalt-type building blocks with alternating cation (GeSb) and anion (Te) layers. These blocks are stacked along the *c*-axis and periodically separated from each other by intrinsic vacancy layers (van der Waals gaps, vdWg’s) between adjacent Te layers^19^. However, local atomic arrangements in GST alloys are controversial and still under discussion. Even a recent high-resolution STEM study was not fully able to reveal the real structure of GST225 and GST124 lattices since the cation layers were not resolved^20^. In addition, there is a structural similarity in the atomic arrangement between the technological important cubic GST and its trigonal phase. Thus, the aim of this work is to study the local atomic arrangement and lattice distortions in Ge-Sb-Te thin films consisting of layered Ge_2_Sb_2_Te_5_ (GST225), Ge_1_Sb_2_Te_4_ (GST124) and Ge_3_Sb_2_Te_6_ (GST326) crystal structures by using a combination of atomic-resolution aberration-corrected (Cs-corrected) high-angle annular dark-filed scanning transmission electron microscopy (HAADF-STEM) imaging and theoretical image simulation. The stacking sequences in layered GST crystal structures are considered in detail and the favourable sequences are identified and discussed. The approach used here can also be applied to the fast evaluation of stacking sequences in layered GST crystal structures grown by different methods.

## Results and Discussion

### Microstructure of *ex-situ* heated GST thin films

**Figure 1:**
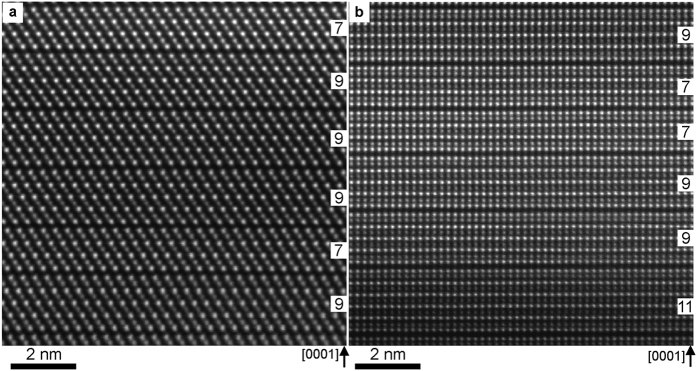
Atomic-resolution Cs-corrected HAADF-STEM images of GST samples prepared at (**a**) 493 K and (**b**) 563 K. The number of layers in each building block is inserted into (**a**,**b**). Viewing direction is 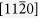 GST in (**a**) and 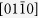 GST in (**b**). The bright dots in (**a,b**) are Te atomic columns whereas the darker dots are GeSb atomic columns. The dark lines are vdWg’s. The GST building blocks are stacked along the c-axis.

The averaged composition of GST thin films was verified by STEM-EDX to be 20 ± 1 at.% of Ge, 24 ± 1 at.% of Sb and 56 ± 1 at.% of Te. Thus, the formation of building blocks with different local composition can be expected in the produced thin films which is in agreement with the equilibrium phase diagram Ge-Sb-Te^21^. The microstructure of GST thin films heated at 493 K and 563 K is shown in Fig. 1a,b, respectively. The thin films consist of different building blocks with 7, 9 and 11 layers, indicating pronounced stacking disorder in the films. The formation of GST124 (with 7 layers) and GST225 (with 9 layers) building blocks was observed in the film heated at 493 K (Fig. 1a) whereas the formation of GST124, GST225 and GST326 (with 11 layers) building blocks was found in the GST films heated at 523 K and 563 K (Fig. 1b). Neither the orientation of the grain towards the substrate (amorphous interface) nor the grain size appears to possess a significant influence on the chemical disorder. Consequently, stacking disorder appears to be typical for layered GST225 compounds^22^. It is worth mentioning that no chemical disorder was reported for the trigonal GST124 phase^20^,^23^. The stacking disorder in the GST225 phase is attributed to deviations in local chemical composition of GST thin films. It is known that the metastable (cubic) GST225 phase is formed before the formation of stable (trigonal) GST225 phase. In the cubic GST225 crystal structure, the Ge, Sb and vacancies are randomly distributed with different Ge/Sb ratios per cation sites^24^. The transition from cubic to trigonal phase begins by ordering of vacancies into vacancy layers separated by GST building blocks containing 4 and 5 Te layers^25^. The number of Te layers depends on local concentration of Ge and Sb in the parent cubic phase, favouring the formation of either GST124 or GST225 building blocks during the phase transition. At higher temperatures or longer annealing time, the formation of GST326 building blocks is expected to compensate the presence of GST124 building blocks in order to retain the overall composition close to GST225 as was already discussed in ref. 22. However, at higher temperatures the formation of GST225 phase containing exclusively GST225 building blocks is also possible since the system is closer to thermodynamic equilibrium. Interestingly, such a GST grain containing only GST225 building blocks was also found in the film heated at 523 K (see Fig. S1 in the Supplementary Information), confirming the previous conclusion. Consequently, a small local chemical gradient and non-equilibrium growth conditions can result in pronounced stacking disorder, as was also observed during synthesis of GST225 nanostructured materials^20^,^26^.

### Local atomic arrangement in GST225 lattice

**Figure 2:**
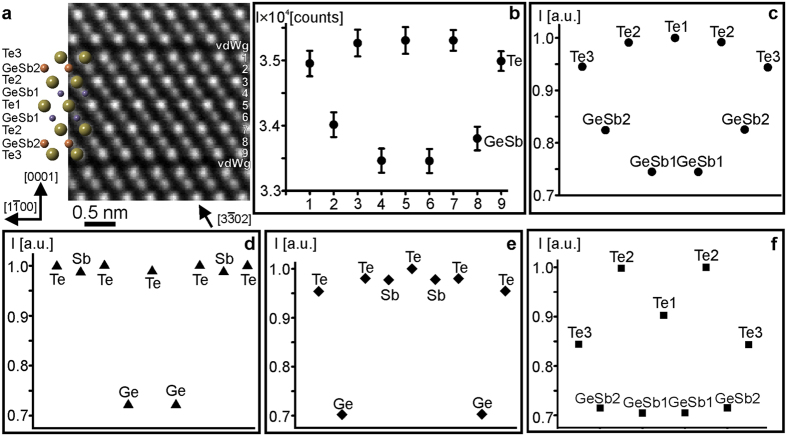
(**a**) Atomic-resolution Cs-corrected HAADF-STEM images of GST225 lattice in 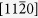 viewing direction. The insert in (**a**) is a schematic representation of the GST225 lattice. **(b)** Experimental averaged intensity maxima from odd numbered rows in (**a**) containing Te and even numbered rows containing GeSb, extracted from an experimental image containing a GST225 building block. Normalized intensity maxima taken from simulated images using GST225 crystal structures with (**c**) Urban sequence^19^, (**d**) Kooi sequence^22^, (**e**) Petrov sequence^32^ and (**f**) Matsunaga sequence^33^.

Experimental atomic-resolution Cs-corrected HAADF-STEM micrograph of the GST225 crystal lattice is given in Fig. 2a. The micrograph represents direct information on the atomic arrangement in the lattice since the HAADF image intensities are proportional to the atomic number approximately according to ~Z^1.75±0.05^ for the inner detector angle of 80 mrad^27^,^28^,^29^,^30^. In addition, the information on the local structure and chemical composition can be obtained by evaluation of image intensities. However, quantitative matching of experimental images with the simulated micrographs is needed for appropriate data interpretation^31^. Consequently, the comparisons between experimental (Fig. 2b) and theoretical averaged intensity maxima for specific lattice sites (Fig. 2c–f) in a GST225 lattice are shown below.

Four structural models for the trigonal GST225 phase are proposed in the literature^19^,^22^,^32^,^33^. In models reported by Petrov *et al.*^32^ and Kooi *et al.*^22^, the cation layers are fully occupied either by Ge or Sb atomic species resulting in the following stacking sequences -Te-vdWg-Te-Ge_1.0_-Te-Sb_1.0_-Te-Sb_1.0_-Te-Ge_1.0_-Te-vdWg-Te- and -Te-vdWg-Te-Sb_1.0_-Te-Ge_1.0_-Te-Ge_1.0_-Te-Sb_1.0_-Te-vdWg-Te-, respectively. The thermal displacement parameters (B) for Te, Ge and Sb in the Petrov and Kooi models were not identified. Thus, for simulation purposes they were equally set to 0.5 Å^2^. In models proposed by Matsunaga *et al.*^33^ and Urban *et al.*^19^, the cation layers are partially occupied by Ge and Sb atoms forming the following stacking sequences -Te3-vdWg-Te3-Ge_0.44_Sb_0.56_2-Te2-Ge_0.56_Sb_0.44_1-Te1-Ge_0.56_Sb_0.44_1-Te2-Ge_0.44_Sb_0.56_2-Te3-vdWg-Te3- and -Te3-vdWg-Te3-Ge_0.33_Sb_0.66_2-Te2-Ge_0.60_Sb_0.36_1-Te1-Ge_0.60_Sb_0.36_1-Te2-Ge_0.33_Sb_0.66_2-Te3-vdWg-Te3-, respectively. The B parameters were experimentally determined for both models (Table 1) and thus, are not equal at different lattice sites. The simulated intensity maxima for the cation and anion sites using different structural models are depicted in Fig. 2c–f. The best match between the simulated and experimental image intensity maxima for the GeSb and Te sites was obtained using the Urban model as the input structure for the simulations which is also supported quantitatively by the cross-correlation coefficients (Kcc) summarized in Table 2. It should be noted that the crystal structures proposed by Matsunaga *et al.*^33^ and Urban *et al.*^19^ are very similar as also evident in the K_cc_ coefficients. The major difference between the models can be found in the B factors at particular Te sites. The B parameters of Te1 and Te2 are different in the Matsunaga model, whereas the B factors of Te1 and Te2 are similar in the Urban model. The observed difference in the simulated intensities at these Te sites in both models can be explained as follows.

The image intensities in HAADF-STEM micrographs are strongly affected by thermal diffuse scattering (TDS) of electrons^34^,^35^,^36^,^37^,^38^. The TDS intensity contribution can be expressed as I_TDS_≈f^ 2^(1-exp(-2DWF)), where f is the atomic scattering factor and DWF is the Debye-Waller factor. Thus, the TDS is rather sensitive to the DWF^34^,^37^,^38^. The latter is defined as DWF = exp(−Bs^2^), where s is the scattering vector. A smaller B factor and hence a larger DWF will result in a stronger thermal scattering and thus a higher intensity in the HAADF image from a specific element^34^. The intensities in simulated HAADF image at the Te1 and Te2 sites using the Matsunaga model are, therefore, different. On the other hand, the intensities at the Te1 and Te2 sites in the simulated image using the Urban model are similar, which is in agreement with the intensities at the Te1 and Te2 sites in the experimental image. The B factor of Te3 in the Matsunaga and Urban models is larger than for the Te1 and Te2. This yields lower intensity at the Te3 site. Furthermore, the intensities at the intermixed GeSb sites in the Matsunga model are mainly dominated by the relative amount of strong scattering Sb with higher number Z (Z_Sb_ (51)> Z_Ge_(32)) since the B factors at these sites are very similar. In the Urban model, the B factor of GeSb2 is smaller than for GeSb1 whereas the relative amount of strong scatter Sb at the GeSb2 site is higher than at the GeSb1 site. These two factors result in higher intensity at the GeSb2 site. It is worth noting that the vicinity of the Te atomic columns to the Ge-rich columns can influence the scattering intensity of Te more than the vicinity of Te to the Sb-rich columns due to the crosstalk between channelled states of the adjacent columns^29^ (see e.g. Fig. 1e). However, the vicinity of Te to the vacancy gap and Sb-rich layers seemingly does not affect the intensity of Te much (see e.g. Fig. 1d).

### Local atomic arrangement in GST124 lattice

**Figure 3:**
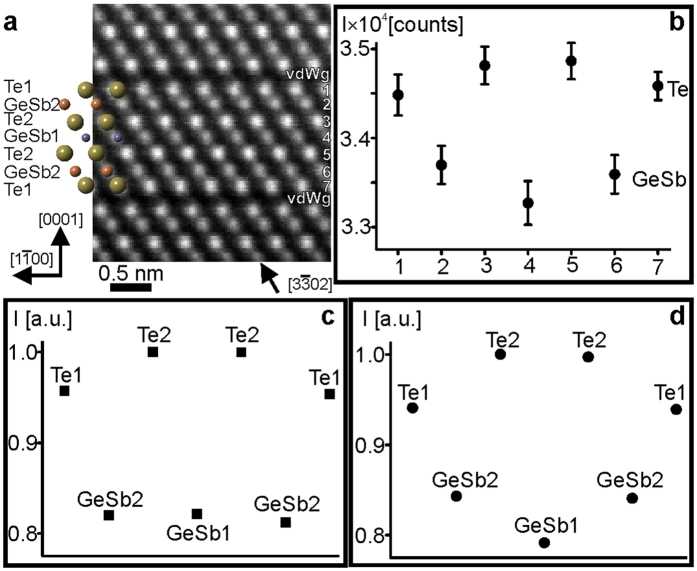
(**a**) High-resolution Cs-corrected HAADF-STEM micrograph of GST124 lattice in 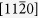 viewing direction. The insert in (**a**) is schematic representation of the GST124 lattice. (**b**) Experimental averaged intensity maxima for Te (odd numbers) and GeSb atomic rows (even numbers) extracted from experimental image containing GST124 building block. Normalized intensity maxima extracted from simulated images using GST124 crystal structures with (**c**) Matsunaga sequence^41^ and (**d**) Karpinsky sequence^40^.

The structural model for GST124 was first proposed by Agaev *et al.*^39^ and then refined by Karpinsky *et al.*^40^, Matsunaga *et al.*^41^ and Sun *et al.*^42^. It should be noted that Sun *et al.*^42^ studied the atomic arrangement in cubic GST124 crystal structure. However, the ab-initio density functional theory (DFT) calculations were performed using GST124 structure with ordered Ge and Sb layers similar to the Kooi model^22^ but with 21R-type stacking sequence (a 21 layer cubic close-packed structure). The stacking sequence proposed by Agaev *et al.*^39^ is -Te_.1.0_-vdWg-Te_1.0_-Sb_1.0_-Sb_1.0_-Te_1.0_-Te_1.0_-Ge_1.0_-Te_1.0_-vdWg-Te_1.0_-. The B parameters were not reported by Sun *et al.*^42^ and Agaev *et al.*^39^ and were set to 0.5 Å^2^ for image simulations. Thus, the scattered intensities in both crystal structures are dominated by the Z number and only small differences in the intensity maxima between Te and Sb tomic columns are expected. The results of image simulations are shown in the Supplementary Information (Fig. S2). In the Karpinsky and Matsunaga models, the cation layers are mixed GeSb layers forming the following stacking sequences -Te1-vdWg-Te1-Ge_0.26_Sb_0.72_2-Te2-Ge_0.43_Sb_0.57_1-Te2-Ge_0.26_Sb_0.72_2-Te1-vdWg-Te1- and -Te1-vdWg-Te1-Ge_0.25_Sb_0.75_2-Te2-Ge_0.49_Sb_0.51_1-Te2-Ge_0.25_Sb_0.75_2-Te1-vdWg-Te1-, respectively. The B factors for both models are listed in Table S1 in the Supplementary Information. Figure 3a represents an experimental atomic-resolution Cs-corrected HAADF-STEM micrograph of the trigonal GST124 crystal lattice whereas Fig. 3b gives experimental averaged intensity maxima for Te and GeSb atomic rows. The intensity maxima for Te and GeSb sites extracted from simulated images for the Matsunaga and Karpinsky models are depicted in Fig. 3c,d, respectively. The simulated image intensities of Fig. 3d best fit the intensity maxima of the experimental image (Fig. 3b). The K_cc_ coefficient between the experimental and simulated image intensities using Karpinsky model supports this conclusion well (see Table 3). It is worth noting that the structural models proposed by Karpinsky *et al.*^40^ and Matsunaga *et al.*^41^ are very similar as is also apparent from the K_cc_ in Table 3. In both structures, the B factor of Te2 is smaller than for Te1, resulting in higher intensity at the Te2 site than at the Te1 site. However, the major difference between the models lies in the B factors at different GeSb sites. The B factor of GeSb2 (1.11 Å^2^) is smaller than for GeSb1 (1.39 Å^2^) in the Karpinsky model whereas the B parameter of GeSb2 (5.23 Å^2^) is larger than for GeSb1 (4.08 Å^2^) in the Matsunaga model. In addition, the occupancy of Sb at the GeSb2 site is higher than at the GeSb1 site in both models. Thus, following the above consideration, the intensity at the GeSb2 sites should be higher than at the GeSb1 site in the Karpinsky model, whereas the intensity at the GeSb2 site has to be equal to the GeSb1 site in the Matsunaga model, as was also observed experimentally. The relative intensity at the GeSb1 site, therefore, is a good indicator for the selection of the best model for the GST124 crystal structure.

### Local atomic arrangement in GST326 lattice

**Figure 4:**
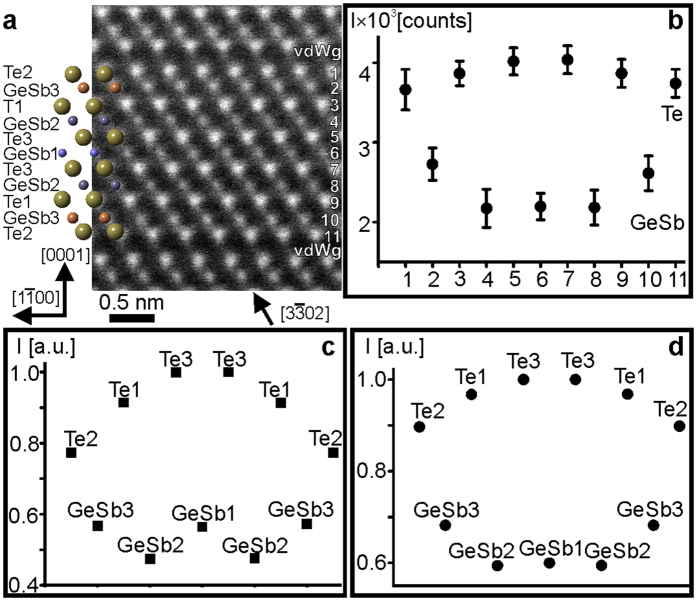
(**a**) Atomic-resolution Cs-corrected HAADF-STEM image of GST326 lattice in 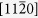 viewing direction. The insert in (**a**) is schematic representation of the GST326 lattice. (**b**) Experimental averaged background subtracted intensity maxima for Te (odd numbers) and GeSb atomic rows (even numbers) extracted from experimental image containing GST326 building block. Normalized background subtracted intensity maxima extracted from simulated images using GST326 crystal structures with (**c**) Matsunaga sequence^43^ and (**d**) Schneider sequence^44^.

Three structural models for GST326 phase are proposed in the literature^42^,^43^,^44^. In the models reported by Matsunaga *et al.*^43^ and Schneider *et al.*^44^, the cation layers are mixed Ge and Sb layers forming the following stacking sequences -Te2-vdWg-Te2-Ge_0.36_Sb_0.64_3-Te1-Ge_0.75_Sb_0.25_2-Te3-Ge_0.77_Sb_0.23_1-Te3-Ge_0.75_Sb_0.25_2-Te1-Ge_0.36_Sb_0.64_3-Te2-vdWg-Te2- and -Te2-vdWg-Te2-Ge_0.55_Sb_0.45_3-Te1-Ge_0.77_Sb_0.23_2-Te3-Ge_0.73_Sb_0.27_1-Te3-Ge_0.77_Sb_0.23_2-Te1-Ge_0.55_Sb_0.45_3-Te2-vdWg-Te2-, respectively. On the other hand, in the model reported by Sun *et al.*^42^, the cation layers are fully occupied either by Ge or Sb atomic species, resulting in the following stacking sequence -Te-vdWg-Te-Sb_1.0_-Te-Ge_1.0_-Te-Ge_1.0_-Te-Ge_1.0_-Te-Sb_1.0_-Te-vdWg-Te-. In the Sun model, the B factors were not reported and were set to 0.5 Å^2^ for all elements. The calculated intensity maxima for GeSb and Te sites for the Sun sequence are depicted in the Supplementary Information (Fig. S3). On the other hand, the B factors given in the Schneider and Matsunaga models have been determined experimentally (see Table S2 in the Supplementary Information). Figure 4a represents an experimental atomic-resolution Cs-corrected HAADF-STEM micrograph of GST326 crystal lattice. Figure 4b shows the experimental averaged background subtracted intensity maxima. Figure 4c,d give intensity maxima for GeSb and Te sites measured from simulated images using the Matsunaga and Schneider models, respectively. The intensity maxima of Fig. 4d match the intensity maxima of the experimental image shown in Fig. 4b well. This conclusion is also supported in a quantitative way by the K_cc_ coefficients summarized in Table 4. The difference between these structural models is also small. The B parameters for various Te sites in both models differ, which therefore results in different intensities at the Te sites. However, the B factors of GeSb in the Schneider model are almost equal, whereas the B parameter of GeSb1 (0.82 Å^2^) in the Matsunaga model is smaller than for the GeSb2 (1.27 Å^2^) and GeSb3 (1.2 Å^2^). Thus, the intensity in the Schneider model are almost equal at the GeSb1 and GeSb2 sites, whereas the intensity at the GeSb1 site in the Matsunaga model is higher than at the GeSb2 site (Fig. 4). The intensity at the GeSb3 site in both models are higher than at the GeSb1 and GeSb2 sites due to the higher occupancy of Sb relative to other GeSb sites.

### Lattice distortions

The building blocks of layered GST crystal structures can be envisaged by the use of (GeSb)Te6 octahedrons. The GeSb atoms in the GST225 structure are off-centre displaced from the centre of these octahedrons in the reported structure models, indicating the distorted octahedral environment of the GeSb cations with strong distortions for the GeSb close to the wdWg and small distortions for the GeSb in the middle of building blocks. The distortions of GST225 lattice can be identified by measuring averaged distances between GeSb and Te atomic columns along the 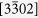 direction. The average distance between Te3 and GeSb2 atomic columns measured from experimental HAADF-STEM images of a GST sample heated at 493 K is 0.204 ± 0.008 nm whereas the distance between GeSb2 and Te2 atomic columns is 0.245 ± 0.007 nm. The average distance between Te2 and GeSb1 atomic columns is 0.212 ± 0.014 whereas the distance between GeSb1 and Te1atomic columns is 0.225 ± 0.014 nm (see Tables S3–S5 for GST samples heated at 523 k and 563 K in the Supplementary Information).

As in GST225, the GeSb atoms in the GS124 structure are displaced from the centre of (GeSb)Te6 octahedrons. However, there are no distortions for the GeSb in the middle of the building blocks in the reported structure models for GST124^40^,^41^. The distance between Te1 and GeSb2 atomic columns in GST124 slabs along the 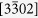 direction measured from experimental HAADF-STEM images of a GST sample heated at 493 K is 0.205 ± 0.009 nm, whereas the distance between GeSb2 and Te2 atomic columns is 0.241 ± 0.007 nm. The Te2-GeSb1 averaged distance is 0.210 ± 0.005 nm, whereas the GeSb1-Te2 distance is 0.226 ± 0.005 nm (see Tables S6–S9 for GST samples heated at 523 K and 563 K in the Supplementary Information).

Similar to the GST124 lattice, the distortions of the (GeSb)Te6 octahedrons in the reported GST326 crystal structures increase from the centre of a building block towards the vdWg where no distortions for the GeSb in the middle of building blocks exist^42^,^44^. The Te2-GeSb3 distance calculated from experimental HAADF-STEM images of a GST sample heated at 523 K is 0.202 ± 0.008 nm, whereas the GeSb3-Te1 distance is 0.247 ± 0.006 nm. The Te1-GeSb2 distance between atomic columns is 0.221 ± 0.016, whereas the distance GeSb2-Te3 is 0.226 ± 0.014 nm. The Te3-GeSb1 distance is 0.225 ± 0.004 nm while the GeSb1-Te3 distance is 0.210 ± 0.004 nm (see Tables S10 and S11 for sample heated at 563 K in the Supplementary Information).

The above results indicate strong distortions for the GeSb located close to the wdWg and small distortions for the GeSb located in the middle of building blocks in all studied crystal structures. Thus, the GeSb atoms are located in distorted octahedral environment with different degree of distortion. Similar lattice distortions were identified for the studied crystal structures produced at 493 K, 523 K and 563 K. Although averaged GeSb-Te distances are in agreement with the above identified GST225, GST124 and GST326 structure models, the local lattice distortions can be larger than in the reported trigonal crystal structures. Notably, the local distortions of (GeSb)Te6 octahedrons in the middle of GST225 (produced at 493 K), GST124 (produced at 493 K and 523 K) and GST326 (produced at 523 K and 563 K) building blocks are more pronounced than in the reported structure models (see Tables S3,S6–S8 and S10–S11 in the Supplementary Information).

The Te-vdWg-Te layer distance is the most crucial factor that affects the band structure of GST225 near the Fermi level. An increased c-parameter in GST225 lattice from 1.725 nm to 1.85 nm caused a band-gap opening and thus destroyed the conducting interface states^14^. The estimated c-parameter of GST225 prepared at 493 K is 1.752 ± 0.004 nm which is 1.5% larger than in the GST225 crystal structures reported in the literature. On the other hand, the estimated c-parameter of GST225 prepared at 563 K is 1.73 ± 0.01 nm and is very close to the reported value of 1.725 nm^19^. Moreover, the a-parameter of GST225 heated at different temperatures was estimated to be 0.427 ± 0.005 nm and is thus in good agreement with the reported value of 0.422 nm^19^. The a- and c/3-parameters of GST124 prepared at different temperatures were estimated to be 0.424 ± 0.002 nm and 1.376 ± 0.005 nm, respectively, which are very close to the reported values of 0.424 nm and 1.371 nm^40^. The estimated a- and c/3-parameters of GST326 (0.425 ± 0.004 nm and 2.09 ± 0.02 nm, respectively) are also very close to the reported values of 0.419 nm and 2.072 nm^44^.

### General discussion on atomic arrangements in layered GST crystal structures

The above results for three GST phases show that the Ge and Sb atomic species tend to form intermixed cation layers which is in agreement with GST structures solved by X-ray diffraction methods. First-principles calculations predicted that the GST225 crystal structure with intermixed GeSb layers has the lowest energy^45^ which is, however, in contrast to the Sun model derived also from first-principles calculations^46^. On the other hand, other theoretical calculations using the DFT methods showed that the stacking sequences in GST225 with ordered cation layers similar to the Kooi model and intermixed cation layers similar to the Matsunaga or Urban model are the most energetically favourable candidates for GST225^42^,^47^. The energy difference between the GST225 crystal structures with ordered and intermixed cation layers is very small^37^. However, the relative energies for both variant were strongly dependent on the choice of the exchange and correlation function. Moreover, no splitting in the Ge–Te bond lengths was found in the GST structures with ordered cation layers (Kooi sequence)^47^. Thus, the ordering of Ge and Sb on cation sites results in the GST structure with non-off-centre displaced Ge within GeTe6 octahedrons, which is inconsistent with the distortions of (GeSb)Te6 octahedrons in the identified GST225 structure as reported by Urban *et al.*^19^ or Matsunaga *et al.*^33^ as well as in the present work.

The crystal structures with ordered and intermixed cation layers were calculated to be the most stable structures for GST124 and GST326, respectively^42^,^45^. However, ordering of Ge cations in GST326 in the middle of building block was assumed. Although different GST compounds (stable and metastable) are very close in terms of structure^17^,^45^,^48^,^49^,^50^,^51^, it is not clear why the cation layers in the GST124 structure should not possess intermixing. As an explanation, it was proposed that the Te, Sb and Ge atoms in the (0001) plane have to satisfy the 3Ge-Te-3Sb rule, in which Te atoms are surrounded by three Ge and three Sb atoms located in opposite corners of the octahedron structures^45^. This, however, does not match the experimental results^40^,^41^. An explanation could be that the theoretical calculations do not take into account the influence of temperature on cation intermixing. Furthermore, the transformation mechanism between metastable and stable GST phases is considered to occur by preferential ordering of vacancies randomly distributed in the metastable GST into vacancy layers without long range diffusion of Ge and Sb and without pronounced change in local composition at cation sites of the parent metastable GST phase^24^,^50^. It was also shown that the layered GST225 structure with intermixed cation layers is thermodynamically stable up to the melting point^19^. In addition, a further reason brought forward for the formation of intermixed cation layers in GST compounds is the higher oxidation state of Sb(III) compared to Ge(II)^19^. This leads to a more favourable charge balance in the coordination sphere of 3-fold coordinated Te next to the wdWg. Thus, the formation of ordered GST structures at elevated temperatures is unfavorable, which might also hinder the synthesis of iPCMs with periodically layered yet chemically distinct structures since the growth of iPCMs requires substrate temperatures comparable with the lowest annealing temperature used in this work.

## Conclusions

The local structure of Ge-Sb-Te phase change thin films was studied by using a combination of atomic-resolution Cs-corrected HAADF-STEM and theoretical image simulation methods. The thin films show pronounced stacking disorder with local formation of layered GST225, GST124 and GST326 building blocks. Sensitivity of HAADF image intensities to B factors in the studied crystal structures at specific lattice sites made it possible to assess experimentally the local atomic arrangements and to identify favourable stacking sequences in three layered GST compounds. The here applied approach can also be used for the evaluation of local atomic arrangements in various layered GST crystal structures by HAADF-STEM. In addition, local lattice distortions in the studied crystal structure are found to be larger than in the literature reported structures. Consequently, the crystal structure of a single GST building block is conceptually similar to the local structure of distorted cubic GST lattice.

The results of this work shed new insight into the local structure of trigonal Ge-Sb-Te compounds. As these can be formed during iPCMs synthesis, a re-evaluation of the possible intermixing effects in layered PCM superstructures and comparison with the here presented stacking-disordered mixed lattices may promote a better future understanding of switching mechanism of iPCMs by theoretical calculations.

## Methods

Amorphous thin films of GST were deposited by pulsed laser deposition (PLD) onto a Si(001)/SiOx substrate^25^. The amorphous GST films were annealed in a vacuum oven at T = 493 K (20 min), T = 523 K (30 min) and T = 563 K (30 min).

Cross-sectional TEM specimens were prepared by a combination of focused Ga high- and Ar low-energy ion beam milling^25^,^52^. The final TEM specimen thickness was 35 ± 7 nm (as measured by electron energy loss spectroscopy).

The atomic-resolution STEM investigations were performed on a probe Cs-corrected Titan^3^ G2 60–300 microscope operating at 300 kV accelerating voltage. A probe forming aperture of 25 mrad (for imaging of GST124 and GST225 crystal lattices) and of 20 mrad (for imaging of GST326 crystal structure) was used in the experiments. All images in this work were acquired with a HAADF (Fischione) detector using annular ranges of 80–200 mrad. The Cs value was tuned to be <200 nm. The C5 parameter was adjusted by the manufacturer to be ≈400 μm. Beam currents were limited to about 80 pA. In order to reduce the influence of the electron beam on the possible randomization of Ge and Sb atomic species, dwell times per pixel of 15 μs for 1 k × 1 k image size and 6 μs for 2 k × 2 k image size were used.

Theoretical image simulations were performed with the xHREM/STEM software based on the absorptive potential approach in the FFT multislice formalism^34^. The effective source size was set to 0.07 nm which corresponds to the specified spatial resolution of the STEM instrument as identified from Fast Fourier Transform (FFT) image calculated from a high-resolution STEM micrograph of the Si [110] substrate.

Analysis of intensity maxima on different lattice sites in the studied structures was performed by detailed peak fitting using the Origin software. At least 60 measurement points per individual layer taken from 10 building blocks of GST225, 10 building blocks of GST124 and 3 building blocks of GST326 were used for the analysis. The intensity maxima were evaluated using unfiltered raw image data. The averaged intensity maxima for different GeSb and Te atomic rows were extracted from intensity profiles taken from experimental and simulated images along the 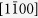 direction. Quantitative comparisons between the experimental and the simulated image intensities were carried-oud by using the Kcc coefficients^53^,^54^.

The real-space structure of GST lattices was directly evaluated from atomic-resolution STEM images. The interatomic distances were calculated by detailed fitting and evaluation of intensity profiles using the Origin software. In order to calculate the interatomic distances, the STEM images were processed by a radial difference filter for noise reduction. The filter is available from HREM Research Inc. as a plug-in for the Gatan Digital Micrograph software suite. It is worth noting that typical specimen drift of the Titan microscope is less than 0.45 nm/min and the spot drift is 0.03 nm/min. This demonstrates that the instrument itself and the ambient room are highly stabilized. Since the specimen drift cannot be entirely eliminated, the correction of residual image distortions was performed using image post-processing with the Jitterbug software (HREM Research Inc.)^55^.

The chemical composition of GST thin films was verified by EDX mapping at different specimen regions using a Super-X EDX detector. EDX spectra were recorded in STEM mode using fast chemical mapping and subsequent averaging the EDX maps. The maps were acquired and evaluated using the Bruker Esprit software.

## Additional Information

**How to cite this article**: Lotnyk, A. *et al.* Local atomic arrangements and lattice distortions in layered Ge-Sb-Te crystal structures. *Sci. Rep.* **6**, 26724; doi: 10.1038/srep26724 (2016).

## Supplementary Material

Supplementary Information

## Figures and Tables

**Table 1:** Thermal displacement parameters (B) for the trigonal GST 225 crystal structures.

Element	B [Å^2^], Matsunaga *et al.*^33^	B [Å^2^], Urban *et al*.^19^
Te1	1.16	1.34
Te2	0.67	1.37
Te3	1.47	1.65
GeSb1	1.8	2.2
GeSb2	1.87	2.04

**Table 2:** Calculated K_cc_ coefficients between experimental and simulated image intensities of GST 225 with different stacking sequences.

	Urban *et al.*^19^	Matsunaga *et al.*^33^	Kooi *et al.*^22^	Petrov *et al.*^32^
K_cc_ (I_raw_)	0.990	0.918	0.757	0.417
σ	0.004	0.008	0.016	0.023

**Table 3:** Calculated K_cc_ coefficients between experimental and simulated image intensities of GST 124 with different stacking sequences.

	Karpinsky *et al.*^40^	Matsunaga *et al.*^41^	Sun *et al.*^42^	Agaev *et al.*^39^
K_cc_ (I_raw_)	0.986	0.961	0.719	0.351
σ	0.005	0.009	0.022	0.044

**Table 4:** Calculated K_cc_ coefficients between the experimental and the simulated image intensities of GST 326 with different stacking sequences.

	Schneider *et al.*^44^	Matsunaga *et al.*^43^	Sun *et al.*^42^
K_cc_ (I_raw_)	0.957	0.932	0.767
σ	0.001	0.002	0.022
