# Supplementary material for: Local atomic arrangements and lattice distortions in layered Ge-Sb-Te crystal structures
Source: Sci Rep. 2016 May 25;6:26724. doi: 10.1038/srep26724 (PMC4879703; doi:10.1038/srep26724)
Supplement: Supplementary Information [file srep26724-s1.doc]

**Supplementary Information**

**Local atomic arrangements and lattice distortions in layered Ge-Sb-Te crystal structures**

**Andriy Lotnyk,^1^,* Ulrich Ross^1^, Sabine Bernütz^1^, Erik Thelander^1^, Bernd Rauschenbach^1^**

^1^Leibniz Institute of Surface Modification (IOM), Permoserstr. 15, D-04318 Leipzig, Germany

* Corresponding author: e-mail: andriy.lotnyk@iom-leipzig.de


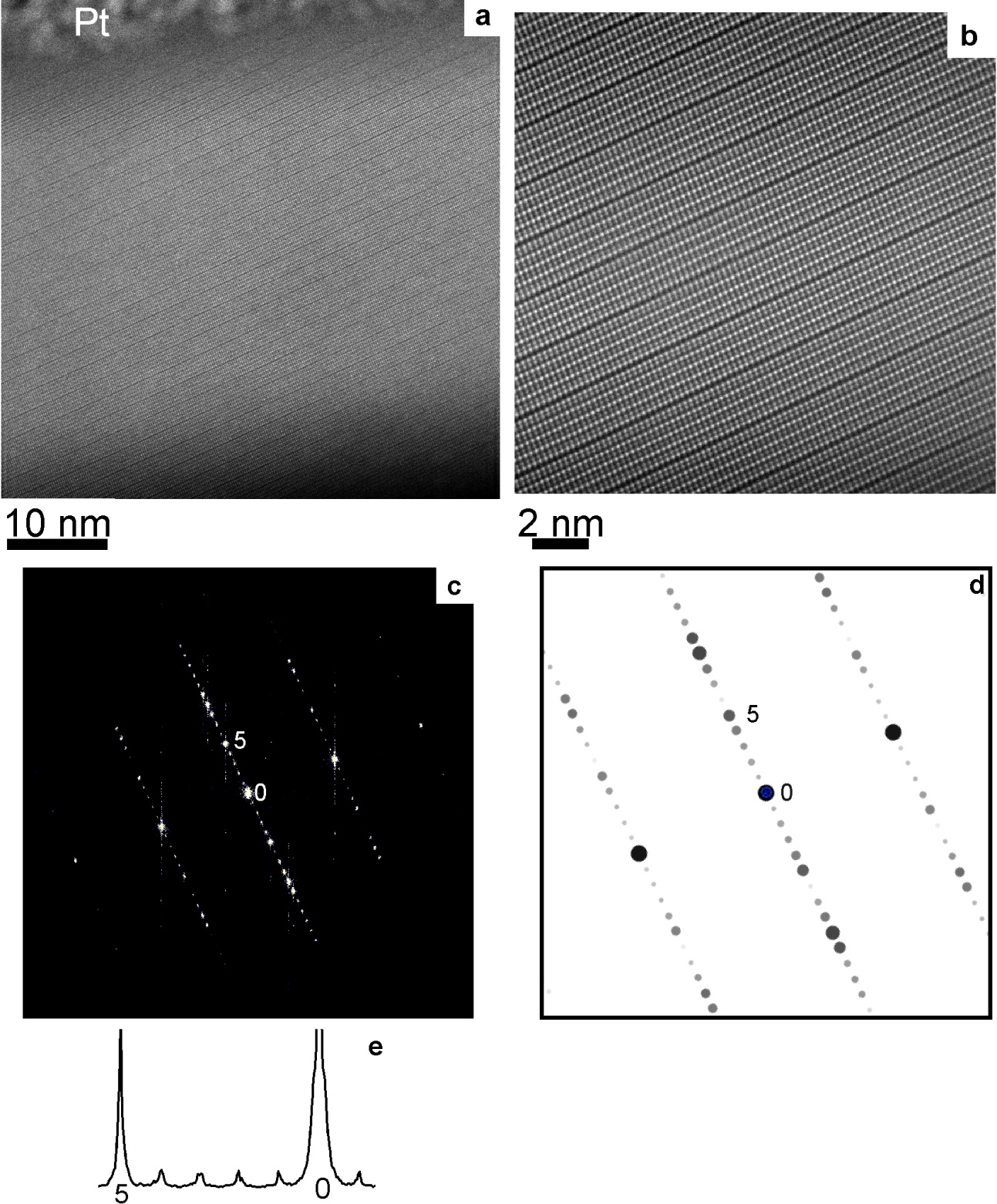


**Figure S1**. (a)-(b) HAADF-STEM images of a grain containing GST225 building blocks observed in the sample prepared at 523 K. (c) Fourier transform image (power spectrum) calculated from whole image (a). (d) Simulated electron diffraction pattern for GST225 based on 2 beam condition (specimen thickness 10 nm). (e) Line profile taken from image (c) along the 0-5. Four spots are located between the 0 and 5 in image (c) and image (d). Viewing direction is $\text{[01}\overline{1}\text{0]}$ GST225. The Pt layer in (a) is due to FIB specimen preparation.

**
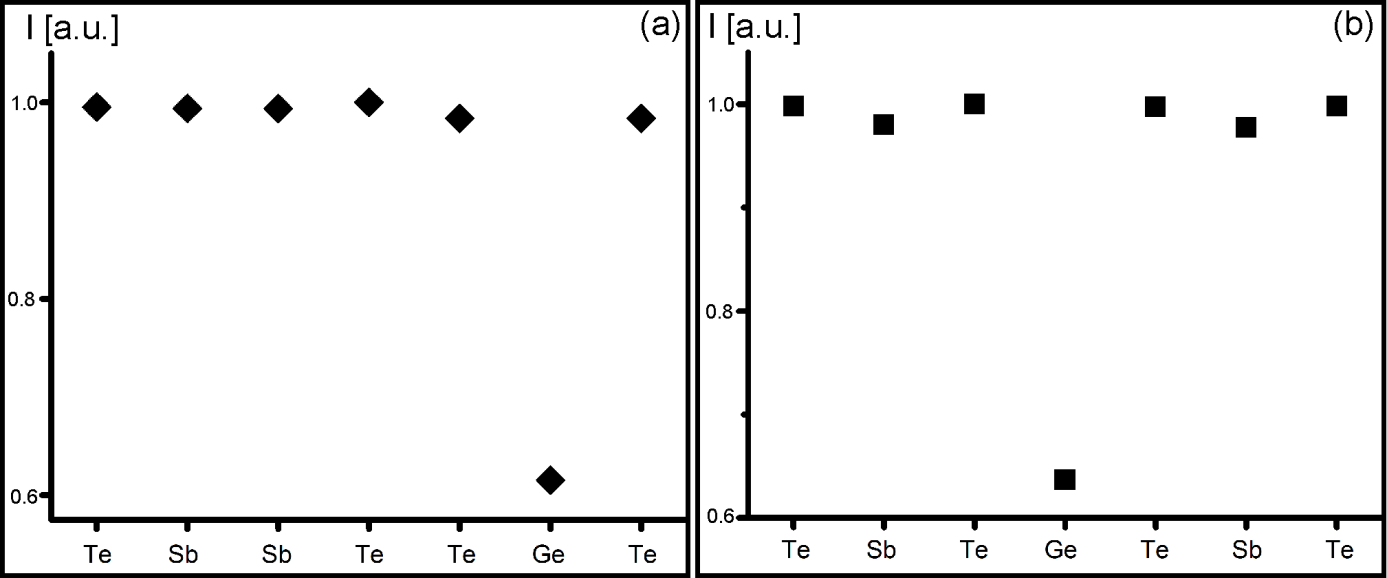
**

**Figure S2**. Normalized background subtracted intensity maxima extracted from simulated images using GST124 crystal structure with (a) Agaev sequence and (b) Sun sequence.


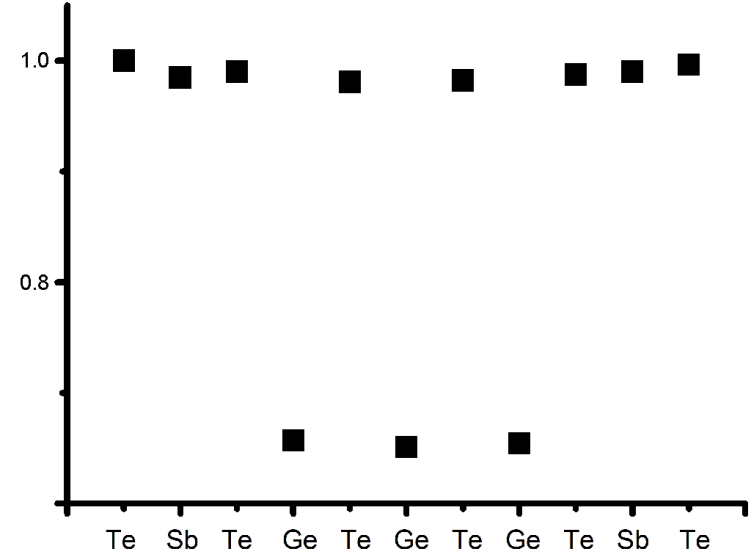


**Figure S3**. Normalized background subtracted intensity maxima extracted from simulated images using GST326 crystal structure with Sun sequence.

| Element | B [Å^2^],  Karpinsky et al.^39^ | B [Å^2^],  Matsunaga et al.^40^ |
| --- | --- | --- |
| Te2 | 0.76 | 2.94 |
| Te1 | 0.95 | 3.4 |
| GeSb1 | 1.39 | 4.08 |
| GeSb2 | 1.11 | 5.23 |

**Table S1.** Thermal parameters (B) parameters for the trigonal GST 124 crystal structures.

| Element | B [Å^2^],  Matsunaga et al.^43^ | B [Å^2^],  Schneider et al.^44^ |
| --- | --- | --- |
| Te1 | 0.51 | 1.88 |
| Te2 | 0.82 | 2.18 |
| Te3 | 0.39 | 1.8 |
| GeSb1 | 0.82 | 2.68 |
| GeSb2 | 1.27 | 2.57 |
| GeSb3 | 1.2 | 2.37 |

**Table S2.** Thermal parameters (B) for the trigonal GST326 crystal structures.

| Projected distance | Te3-GeSb2 | GeSb2-Te2 | Te2-GeSb1 | GeSb1-Te1 | Te1-GeSb1 | GeSb1-Te2 | Te2-GeSb2 | GeSb2-Te3 |
| --- | --- | --- | --- | --- | --- | --- | --- | --- |
| Column notation | L(1) | (L2) | L(3) | L(4) | L(4) | R(3) | (R2) | R(1) |
| Measurement [nm] | 0,198 | 0,249 | 0,200 | 0,237 | 0,213 | 0,225 | 0,242 | 0,21 |
| σ | 0,006 | 0,007 | 0,005 | 0,005 | 0,006 | 0,007 | 0,006 | 0,005 |
| Average value of L(X) & R(X) [nm] | 0,204 | 0,245 | 0,212 | 0,225 |  |  |  |  |
| σ | 0,008 | 0,007 | 0,014 | 0,014 |  |  |  |  |
| Literature value [nm]^19^ | 0,202 | 0,242 | 0,209 | 0,221 | 0,221 | 0,209 | 0,242 | 0,202 |

**Table S3.** Average Te-GeSb distances measured for GST225 building blocks. The GST sample was prepared by heating at 493 K. The line profiles (total 30; 5 profiles per one GST225 block) were taken along $\text{[3}\overline{3}\text{02]}$GST direction of a STEM image acquired along $\text{[11}\overline{2}\text{0]}$GST viewing direction as shown in Fig. 1a.

| Projected distance | Te3-GeSb2 | GeSb2-Te2 | Te2-GeSb1 | GeSb1-Te1 | Te1-GeSb1 | GeSb1-Te2 | Te2-GeSb2 | GeSb2-Te3 |
| --- | --- | --- | --- | --- | --- | --- | --- | --- |
| Column notation | L(1) | (L2) | L(3) | L(4) | L(4) | R(3) | (R2) | R(1) |
| Measurement [nm] | 0,163 | 0,217 | 0,176 | 0,193 | 0,192 | 0,176 | 0,215 | 0,164 |
| σ | 0,006 | 0,009 | 0,008 | 0,008 | 0,010 | 0,008 | 0,008 | 0,009 |
| Average value of L(X) & R(X) [nm] | 0,163 | 0,216 | 0,176 | 0,193 |  |  |  |  |
| σ | 0,008 | 0,008 | 0,008 | 0,009 |  |  |  |  |
| Literature value [nm]^19^ | 0,160 | 0,209 | 0,170 | 0,184 | 0,184 | 0,170 | 0,209 | 0,160 |

**Table S4.** Average Te-GeSb distances measured for GST225 building blocks. The GST sample was prepared by heating at 523 K. The line profiles (total 35; 7 profiles per one GST225 block) were taken along [0001] GST direction of a STEM image recorded along $\text{[01}\overline{1}\text{0]}$ GST viewing direction as shown in Fig. 1b.

| Projected distance | Te3-GeSb2 | GeSb2-Te2 | Te2-GeSb1 | GeSb1-Te1 | Te1-GeSb1 | GeSb1-Te2 | Te2-GeSb2 | GeSb2-Te3 |
| --- | --- | --- | --- | --- | --- | --- | --- | --- |
| Column notation | L(1) | (L2) | L(3) | L(4) | L(4) | R(3) | (R2) | R(1) |
| Measurement [nm] | 0,161 | 0,205 | 0,172 | 0,184 | 0,184 | 0,173 | 0,205 | 0,165 |
| σ | 0,005 | 0,003 | 0,003 | 0,005 | 0,005 | 0,007 | 0,006 | 0,008 |
| Average value of L(X) & R(X) [nm] | 0,163 | 0,205 | 0,172 | 0,184 |  |  |  |  |
| σ | 0,007 | 0,004 | 0,005 | 0,005 |  |  |  |  |
| Literature value [nm]^19^ | 0,160 | 0,209 | 0,170 | 0,184 | 0,184 | 0,170 | 0,209 | 0,160 |

**Table S5.** Average Te-GeSb distances measured for GST225 building blocks. The GST sample was prepared by heating at 563 K. The line profiles (total 5; 5 profiles per one GST225 block) were taken along [0001] GST direction of a STEM image recorded along $\text{[01}\overline{1}\text{0]}$GST viewing direction as shown in Fig. 1b.

| Projected distance | Te1-GeSb2 | GeSb2-Te2 | Te2-GeSb1 | GeSb1-Te2 | Te2-GeSb2 | GeSb2-Te1 |
| --- | --- | --- | --- | --- | --- | --- |
| Column notation | L(1) | L(2) | L(3) | R(3) | R(2) | R(1) |
| Measurement [nm] | 0,200 | 0,245 | 0,211 | 0,227 | 0,237 | 0,215 |
| σ | 0,005 | 0,005 | 0,004 | 0,005 | 0,005 | 0,005 |
| Average value of L(X) & R(X) [nm] | 0,207 | 0,241 | 0,219 |  |  |  |
| σ | 0,009 | 0,007 | 0,009 |  |  |  |
| Literature value [nm]^39^ | 0,204 | 0,239 | 0,216 | 0,216 | 0,239 | 0,204 |

**Table S6.** Average Te-GeSb distances measured for GST124 building blocks. The GST sample was prepared at 493 K. The line profiles (total 45; 5profiles per one GST124 block) were taken along $\text{[3}\overline{3}\text{02]}$GST direction of a STEM image acquired along $\text{[11}\overline{2}\text{0]}$GST viewing direction as shown in Fig. 1a.

| Projected distance | Te1-GeSb2 | GeSb2-Te2 | Te2-GeSb1 | GeSb1-Te2 | Te2-GeSb2 | GeSb2-Te1 |
| --- | --- | --- | --- | --- | --- | --- |
| Column notation | L(1) | L(2) | L(3) | R(3) | R(2) | R(1) |
| Measurement [nm] | 0,212 | 0,237 | 0,225 | 0,212 | 0,248 | 0,198 |
| σ | 0,007 | 0,004 | 0,005 | 0,003 | 0,005 | 0,005 |
| Average value of L(X) & R(X) [nm] | 0,205 | 0,242 | 0,219 |  |  |  |
| σ | 0,009 | 0,007 | 0,008 |  |  |  |
| Literature value [nm]^39^ | 0,204 | 0,239 | 0,216 | 0,216 | 0,239 | 0,204 |

**Table S7.** Average Te-GeSb distances measured for GST124 building blocks. The GST sample was prepared at 523 K. The line profiles (total 22; 4-5 profiles per one GST124 block) were taken along $\text{[3}\overline{3}\text{02]}$GST direction of a STEM image acquired along $\text{[11}\overline{2}\text{0]}$GST viewing direction as shown in Fig. 1a.

| Projected distance | Te1-GeSb2 | GeSb2-Te2 | Te2-GeSb1 | GeSb1-Te2 | Te2-GeSb2 | GeSb2-Te1 |
| --- | --- | --- | --- | --- | --- | --- |
| Column notation | L(1) | L(2) | L(3) | R(3) | R(2) | R(1) |
| Measurement [nm] | 0,175 | 0,205 | 0,197 | 0,175 | 0,222 | 0,159 |
| σ | 0,005 | 0,007 | 0,007 | 0,006 | 0,009 | 0,004 |
| Average value of L(X) & R(X) [nm] | 0,167 | 0,214 | 0,186 |  |  |  |
| σ | 0,009 | 0,009 | 0,0096 |  |  |  |
| Literature value [nm]^39^ | 0,163 | 0,205 | 0,178 | 0,178 | 0,205 | 0,163 |

**Table S8.** Average Te-GeSb distances measured for GST124 building blocks. The GST sample was prepared at 523 K. The line profiles (total 11; 3-4 profiles per one GST124 block) were taken along [0001] GST direction of a STEM image recorded along $\text{[01}\overline{1}\text{0]}$GST viewing direction as shown in Fig. 1b.

| Projected distance | Te1-GeSb2 | GeSb2-Te2 | Te2-GeSb1 | GeSb1-Te2 | Te2-GeSb2 | GeSb2-Te1 |
| --- | --- | --- | --- | --- | --- | --- |
| Column notation | L(1) | L(2) | L(3) | R(3) | R(2) | R(1) |
| Measurement [nm] | 0,164 | 0,207 | 0,179 | 0,179 | 0,205 | 0,167 |
| σ | 0,004 | 0,005 | 0,006 | 0,007 | 0,005 | 0,005 |
| Average value of L(X) & R(X) [nm] | 0,165 | 0,206 | 0,179 |  |  |  |
| σ | 0,005 | 0,005 | 0,006 |  |  |  |
| Literature value [nm]^39^ | 0,163 | 0,205 | 0,178 | 0,178 | 0,205 | 0,163 |

**Table S9.** Average Te-GeSb distances measured for GST124 building blocks. The GST sample was prepared at 563 K. The line profiles (total 58; 4-5 profiles per one GST124 block) were taken along [0001] GST direction of a STEM image recorded along $\text{[01}\overline{1}\text{0]}$GST viewing direction as shown in Fig. 1b.

| Projected distance | Te2-GeSb3 | GeSb3-Te1 | Te1-GeSb2 | GeSb2-Te3 | Te3-GeSb1 | GeSb1-Te3 | Te3-GeSb2 | GeSb2-Te1 | Te1-GeSb3 | GeSb3-Te2 |
| --- | --- | --- | --- | --- | --- | --- | --- | --- | --- | --- |
| Column notation | L(1) | L(2) | L(3) | L(4) | L(5) | R(5) | R(4) | R(3) | R(2) | R(1) |
| Measurement [nm] | 0,208 | 0,243 | 0,215 | 0,219 | 0,225 | 0,210 | 0,233 | 0,204 | 0,250 | 0,196 |
| σ | 0,003 | 0,006 | 0,006 | 0,004 | 0,004 | 0,004 | 0,004 | 0,004 | 0,005 | 0,005 |
| Average value of L(X) and R(X) [nm] | 0,202 | 0,247 | 0,210 | 0,226 | 0,217 |  |  |  |  |  |
| σ | 0,008 | 0,006 | 0,007 | 0,008 | 0,008 |  |  |  |  |  |
| Literature value [nm]^44^ | 0,197 | 0,243 | 0,203 | 0,225 | 0,214 | 0,214 | 0,225 | 0,203 | 0,243 | 0,197 |

**Table S10**. Average Te-GeSb distances measured for GST326 building blocks. The GST sample was prepared at 523 K. The line profiles (total 12; 6 profiles per one GST326 block) were taken along $\text{[3}\overline{3}\text{02]}$GST direction of a STEM image acquired along $\text{[11}\overline{2}\text{0]}$GST viewing direction as shown in Fig. 1a.

| Projected distance | Te2-GeSb3 | GeSb3-Te1 | Te1-GeSb2 | GeSb2-Te3 | Te3-GeSb1 | GeSb1-Te3 | Te3-GeSb2 | GeSb2-Te1 | Te1-GeSb3 | GeSb3-Te2 |
| --- | --- | --- | --- | --- | --- | --- | --- | --- | --- | --- |
| Column notation | L(1) | L(2) | L(3) | L(4) | L(5) | R(5) | R(4) | R(3) | R(2) | R(1) |
| Measurement [nm] | 0,166 | 0,206 | 0,172 | 0,183 | 0,181 | 0,174 | 0,189 | 0,164 | 0,214 | 0,152 |
| σ | 0,008 | 0,007 | 0,005 | 0,007 | 0,006 | 0,005 | 0,006 | 0,005 | 0,005 | 0,004 |
| Average value of L(X) and R(X) [nm] | 0,159 | 0,210 | 0,168 | 0,186 | 0,178 |  |  |  |  |  |
| σ | 0,009 | 0,007 | 0,006 | 0,007 | 0,006 |  |  |  |  |  |
| Literature value [nm]^44^ | 0,155 | 0,211 | 0,163 | 0,190 | 0,176 | 0,176 | 0,190 | 0,163 | 0,211 | 0,155 |

**Table S11.** Average Te-GeSb distances measured for GST326 building blocks. The GST sample was prepared at 563 K. The line profiles (total 11; 11 profiles per one GST326 block) were taken along [0001] GST direction of a STEM image recorded along $\text{[01}\overline{1}\text{0]}$GST viewing direction as shown in Fig. 1b.
